# Transcatheter arterial embolization for unruptured renal angiomyolipoma using a 1.8-Fr tip microballoon catheter with a mixture of ethanol and Lipiodol

**DOI:** 10.1186/s42155-019-0095-8

**Published:** 2020-01-08

**Authors:** Kosuke Tomita, Tomohiro Matsumoto, Shunsuke Kamei, Shota Yamamoto, Satoshi Suda, Hidenori Zakoji, Terumitsu Hasebe

**Affiliations:** 1 Department of Radiology, Tokai University Hachioji Hospital, Tokai University School of Medicine, 1838 Ishikawa-machi, Hachioji, Tokyo, 192-0032 Japan; 2 Department of Urology, Tokai University Hachioji Hospital, Tokai University School of Medicine, 1838 Ishikawa-machi, Hachioji, Tokyo, 192-0032 Japan

**Keywords:** Renal angiomyolipoma, Interventional radiology, Microballoon catheter, Embolization

## Abstract

**Background:**

To evaluate the efficacy and safety of transcatheter arterial embolization for renal angiomyolipoma using a 1.8-French tip microballoon catheter and a mixture of ethanol and Lipiodol.

**Methods:**

Seven consecutive patients with total of eight angiomyolipomas underwent this procedure between June 2014 and June 2017. A 1.8-French tip microballoon catheter was advanced to the feeding artery of the angiomyolipoma, and transcatheter arterial embolization was performed with a mixture of ethanol and Lipiodol under microballoon inflation. We retrospectively evaluated the characteristics of angiomyolipomas, technical success rate, clinical success rate, renal function, and adverse events. Technical success and clinical success were defined as complete embolization of all feeding arteries and reduction of tumor size, respectively.

**Results:**

The median size of the angiomyolipomas was 46 mm (range, 40–64 mm). Transcatheter arterial embolization was successful in all eight angiomyolipomas. The median volume of the mixture of ethanol and Lipiodol was 6.0 ml (range, 2.0–14 ml). The median ratio of ethanol to Lipiodol was 71% (range, 71–75%). All eight angiomyolipomas shrank with a median shrinkage rate of 34% in diameter (range, 9–63%) and 77% in volume (range, 48–94%). The median follow-up period was 13 months (range, 9–54 months). Clinical success was achieved in all cases. Serum creatinine concentrations and the pre- and post-procedural estimated glomerular filtration rates did not change notably, and there were no major complications.

**Conclusion:**

Transcatheter arterial embolization for renal angiomyolipoma using a 1.8-French tip microballoon catheter with a mixture of ethanol and Lipiodol is effective and safe.

## Background

Renal angiomyolipoma (AML) is a neoplasm composed of varying amounts of abnormal blood vessels, smooth muscle, and adipose tissue (Hajdu & Foote Jr., 1969). The main complication of renal AML is retroperitoneal hemorrhage caused by tumor rupture, which can be life-threatening. There are a variety of treatment options for renal AML, such as surgery, transcatheter arterial embolization (TAE), radiofrequency ablation, and mTOR inhibitors. Among them, TAE is the first-line treatment option for both ruptured and unruptured AMLs (Flum et al., 2016; Murray & Lee, 2018). Generally, a tumor larger than 4 cm, tumor with aneurysm larger than 5 mm, and symptomatic renal AML, have been treated using TAE to prevent or treat bleeding (Oesterling et al., 1986; Yamakado et al., 2002). Various kinds of embolic agents are used in TAE for renal AML. While no single embolic agent has been shown to demonstrate superior safety and efficacy, absolute ethanol may be preferred because it can completely occlude the small arteries supplying the AML (Kiefer & Stavropoulos, 2017; Park et al., 1986). Absolute ethanol is often mixed with Lipiodol (Andre Guerbet, Aulnay-Sous-Bois, France), which renders the mixture radiopaque enabling fluoroscopy and prevents reflux of the embolic agent and insufficient embolization. Furthermore, Lipiodol keeps ethanol in blood vessels longer prolonging the reaction time enhancing embolization (Park et al., 1986).

Recently, selective TAE for renal AMLs using a microballoon catheter with a 2.2-French (Fr) tip or bigger used with ethanol was reported to be an effective and safe technique (Sawada et al., 2017). However, the microballoon catheter routinely needs a 5-Fr or larger guiding catheter for TAE. Moreover, sometimes it is difficult to advance the microballoon catheter into the target feeding arteries. In such cases, it is necessary to switch to a microcatheter (Baba et al., 2014; Sawada et al., 2017). The latest microballoon catheter that has been released in 2014 is the 1.8-Fr tip (Matsumoto et al., 2018). This allows advancement of the microballoon catheter coaxially through a 4-Fr angiography catheter. Here we investigated whether the 1.8-Fr tip microballoon catheter could be advanced into the target feeding arteries of renal AML. The aim of this study was to evaluate the efficacy and safety of TAE for unruptured renal AML using the 1.8-Fr tip microballoon catheter with a mixture of ethanol and Lipiodol.

## Methods

### Patients

This retrospective study was conducted with the approval of the Institutional Review Board (17R-303). Written informed consent for the procedure was obtained from each patient. Eight patients with 9 AMLs were entered into the study and underwent TAE between June 2014 and June 2017 in our institution. One patient had emergent TAE for ruptured renal AML and was excluded because in addition to ethanol and Lipiodol we used n-butyl cyanoacrylate and coils as embolic agents. Therefore, the data reported are for 7 patients (2 men, 5 women. Mean age 57 years) with 8 unruptured renal AMLs who underwent TAE using a 1.8-Fr tip microballoon catheter with a mixture of ethanol and Lipiodol (Table 1). The TAE indication criteria for unruptured renal AML in our institution are: tumor larger than 40 mm, tumor with aneurysm larger than 5 mm, or symptomatic tumor according to published literature (Yamakado et al., 2002). The diagnosis of renal AML was made based on dynamic computed tomography (CT) findings. The diagnosis of tuberous sclerosis was made as previously reported (Northrup et al., 2013), and all patients were diagnosed with sporadic renal AML.

**Table 1:** Patient characteristics

Patient No.	Tumor No.	Age	Gender	Symptom	Tuberous sclerosis	Located kidney	Aneurysm
1	1	58	Male	No	No	Left	No
2	2	59	Female	No	No	Left	No
3	3	62	Female	No	No	Right	No
4	4	57	Female	Hematuria	No	Right	Yes
5	5	40	Female	Hematuria	No	Left	Yes
	6			Flank pain		Left	
6	7	70	Male	No	No	Left	Yes
7	8	50	Female	No	No	Right	Yes

*AML* angiomyolipoma, *TAE* transcatheter arterial embolization, *Fr* French, *No.* number

Note: Patient no. 5 had 2 AMLs

### Procedure

**Fig. 1:**
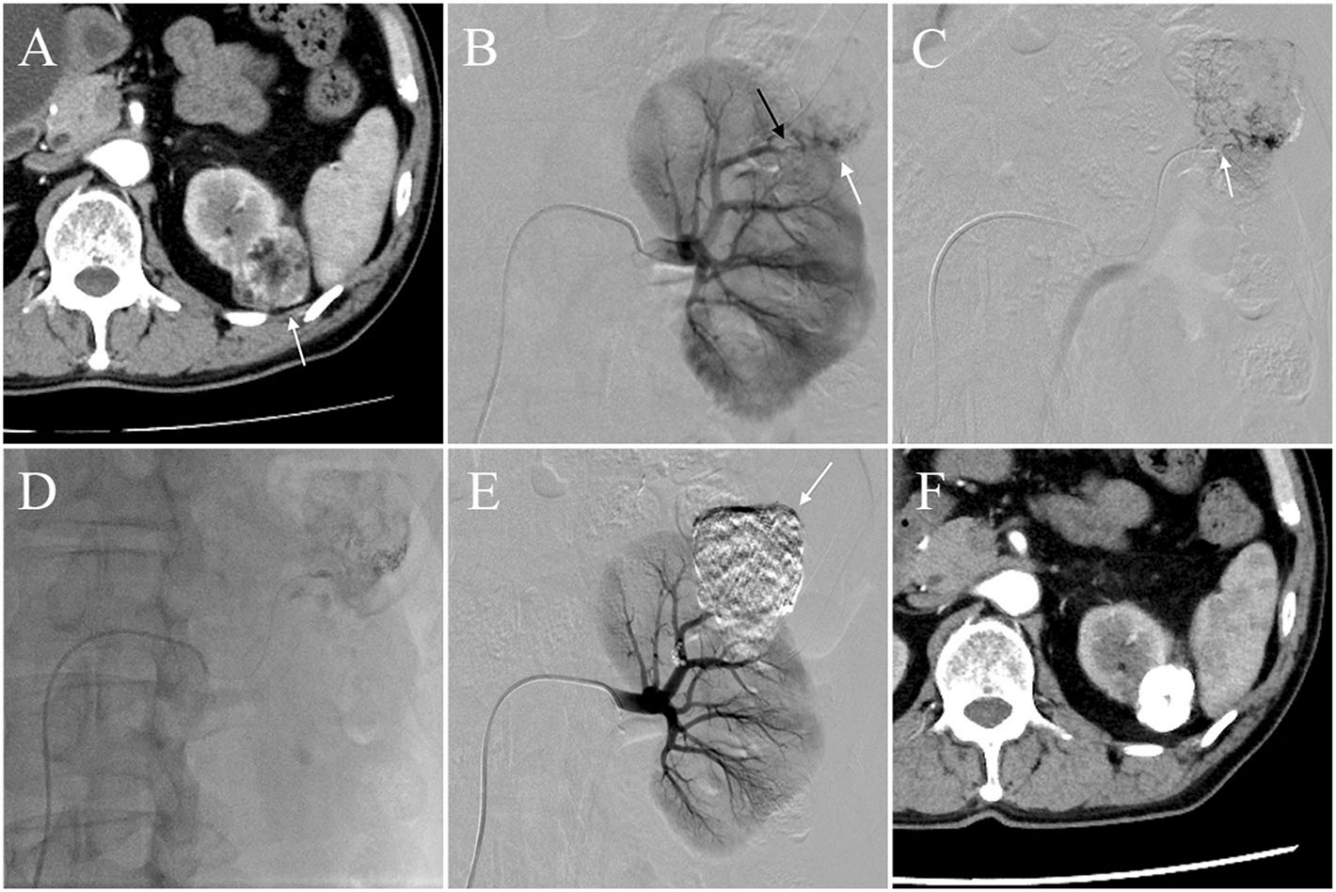
A 70-year-old man with left renal AML (Patient 6); the diameter of AML was 40 mm. **a**. The pre-procedure CT image showing a 40-mm left renal AML (arrow). **b**. Left renal angiography showing a tumor feeding artery (black arrow) and an aneurysm of the AML (white arrow). **c**. 1.8-Fr tip microballoon catheter was advanced into the feeding artery of the tumor (arrow), and angiography showed contrast enhancement of the tumor. **d**. The mixture of the ethanol and Lipiodol was injected under microballoon inflation. **e**. Left renal angiography showing the disappearance of contrast enhancement of the tumor after TAE. The AML, which was filled with Lipiodol and ethanol is shown with arrow. **f**. Follow-up CT after TAE at 10 months showing shrinkage of the tumor and no renal infarcts

In all cases, the right femoral artery was used as the access route. A 4-Fr sheath was inserted into the abdominal aorta via the right common femoral artery under local anesthesia. A 4-Fr catheter was inserted through the 4-Fr sheath, and the renal artery was accessed. Then, renal arteriography was performed to identify the feeding artery of the tumor and to confirm the contrast enhancement of renal AML tumor. Based on the digital subtraction angiography (DSA), the tumors were graded into 3 grades as previously described (Rimon et al., 2006): Grade 1, minimal vascularity: few, small, stretched pathological vessels; Grade 2, moderate vascularity: abundant, medium-size, tortuous vessels with or without small aneurysms (< 5 mm); Grade 3, marked vascularity: multiple, large tortuous vessels with/without aneurysms (> 5 mm). Then, the 1.8-Fr tip microballoon catheter (Attendant Nexus/Occlusafe, Terumo, Tokyo, Japan/Terumo Europe NV, Leuven, Belgium, or Logos ST, Piolax, Yokohama, Japan) was coaxially inserted into the renal AML feeding artery distal to the arteries supplying the normal renal parenchyma. Details regarding each 1.8-Fr tip microballoon catheter are summarized elsewhere (Matsumoto et al., 2018). The choice of 1.8-Fr tip microballoon catheter was made based on physician preference. In all cases, a 0.016-in. guidewire (Asahi Intecc Co., Ltd., Nagoya, Japan) was used to navigate the microballoon catheter. The location of the microballoon and the treatment area were determined using DSA and cone-beam CT, using a flat-panel detector (Siemens Medical Solutions, Forchheim, Germany). After the inflation of the microballoon, DSA was performed again to confirm the tumor enhancement and that non-target blood vessels were spared. Then, a mixture of ethanol and Lipiodol was injected under fluoroscopy. The ratio (v/v) of the mixture was 75% ethanol and 25% Lipiodol, or 71% ethanol and 29% Lipiodol. The choice was made based on physician preference. The injection was continued until the entire AML was filled. After the injection, the microballoon was left inflated for 10 min and then deflated. Renal angiography was performed to reevaluate tumor vascularity. If tumor enhancement was still visualized, the procedure was repeated. Until the feeding artery of the AML disappeared (Fig. 1).

### Parameters investigated

Medical records and procedural images were reviewed to assess technical success rates, clinical success rates, and adverse events (AEs).

The technical success was defined as successful completion of TAE for all feeding arteries by the method described above in the “Procedure” section. Clinical success was defined as tumor shrinkage more than 30% in volume after the procedure. Tumor shrinkage rate was calculated using the length of the major diameter of the largest section in the axial image and tumor volume on CT or magnetic resonance imaging (MRI). Tumor volume was approximated using ellipsoid formula: (width × depth × height) × (π/6). Diameters of the tumor were measured from axial and coronal images on CT or MRI. The diameter of the aneurysm was measured form the axial image on CT. AEs were graded according to the Common Terminology Criteria for Adverse Events version 5.0 (CTCAE v5.0). Serious AEs were defined as AEs of CTCAE grade 3 or higher. Non-serious AEs were defined as CTCAE grade 2 or lower.

### Statistical analyses

Statistical analysis was performed using commercial software (JMP14: SAS Japan, Tokyo, Japan). The Wilcoxon signed-rank test was used to compare pre- and post-procedure measurements. *p* < 0.05 was considered significant.

## Results

### Patient characteristics

The median diameter of renal AML before the procedure was 46 mm (range, 40–64 mm). Aneurysms were found in 5 out of 8 AMLs, and the median size of the aneurysms was 3 mm (range 2–4 mm). Using DSA, 2 tumors were classified as Grade 1, 4 tumors were Grade 2, and 2 tumors were Grade 3.

### Details of the procedures

The details of the procedures are summarized in Table 2. Attendant Nexus and Logos ST microballoon catheters were used in 2 and 5 cases, respectively.

**Table 2:** Details of TAE for renal AML using a 1.8-Fr tip microballoon catheter

Patient No.	Tumor No.	DSA grade (1–3)	Number of feeding artery(s)	Ethanol to Lipiodol ratio (%)	Amount of the mixture (ml)	Amount of ethanol (ml)	Procedure time (min)	Microballoon catheter used
1	1	I	1	75	2.0	1.4	53	Attendant Nexus
2	2	III	2	75	10.0	7.1	104	Attendant Nexus
3	3	I	1	71	6.0	4.3	123	Logos ST
4	4	II	1	71	7.5	5.4	99	Logos ST
5	5	II	1	71	2.7	1.9	89	Logos ST
	6	III	2		2.8	2.0		Logos ST
6	7	II	1	71	14	10.0	53	Logos ST
7	8	II	1	71	6.0	4.3	85	Logos ST

*TAE* transcatheter arterial embolization, *AML* angiomyolipoma, *Fr* French, *No.* number, *DSA* digital subtraction angiography

Technical success was achieved in all cases. Two AMLs had 2 feeding arteries, and the remaining AMLs had 1 feeding artery. The median procedure time was 89 min (range, 53–123 min). The median volume of ethanol-Lipiodol mixture was 6.0 ml (range, 2.0–14 ml) and ethanol: Lipiodol ratio was 71:29 in 5 patients and 75:25 in 2 patients.

### Tumor shrinkage

Clinical success was achieved in all cases. The median follow-up period was 13 months (range, 9–54 months). The median shrinkage in diameter and volume was 34% (range, 9–63%) and 77% (range, 48–94%), respectively (Table 3).

**Table 3:** Comparison of diameter and volume of tumors between pre-TAE and post-TAE

Patient No.	Tumor No.	Long-axis length of the tumor (mm)		Volume of tumor (mm^3^)		Diameter reduction rate (%)	Volume reduction rate (%)	Follow-up period (months)
		Pre- TAE	Post- TAE	Pre- TAE	Post- TAE			
1	1	44	19	35,732	2646	57	93	54
2	2	42	34	30,084	15,452	19	49	14
3	3	55	50	38,013	19,871	9	48	9
4	4	64	24	82,938	5089	63	94	13
5	5	55	24	60,821	4976	56	92	20
	6	49	35	36,637	7660	29	79	
6	7	40	29	22,075	6681	28	70	10
7	8	40	24	20,106	5014	40	75	14

*TAE* transcatheter arterial embolization, *No.* number

Volume of the tumor was calculated with the following formula: (width × depth × height) × (π/6)

### Adverse events

CTCAE Grade 1 back pain was reported in 4 patients, and Grade1 fever in 2 patients. The mean pre- and post-procedure serum creatinine concentrations were all within the normal range (0.77 ± 0.16 and 0.71 ± 0.13 mg/dl, respectively). Pre- and post-procedure estimated glomerular filtration rates (eGFR) were lower than the reference value of 90 ml/min/1.73 m^2^ (66.4 ± 10.6 and 72.5 ± 11.0 ml/min/1.73 m^2^, respectively). There were no significant differences in serum creatinine level (*p* = 0.21) and eGFR (*p* = 0.14) between the pre- and post-procedure values. There were no serious AEs.

## Discussion

The evolution of microballoons have enabled various procedures such as balloon-occluded transarterial chemoembolization for hepatocellular carcinoma (Lucatelli et al., 2019; Matsumoto et al., 2015; Matsumoto et al., 2016; Matsumoto et al., 2017), balloon-occluded retrograde transvenous obliteration for gastric varices (Mine et al., 2017), and coaxial microballoon-occluded coil embolization for vascular disorders (Yasumoto et al., 2015). The present study shows, for the first time, the effectiveness and safety of TAE using a 1.8-Fr tip microballoon catheter with a mixture of ethanol and Lipiodol in the treatment of unruptured renal AML.

In this study, technical success was achieved in all cases. We were able to advance the 1.8-Fr tip microballoon catheter selectively into all target arteries without using a guiding catheter, while 2.2-Fr tip or bigger tip microballoon catheters used with a 5-Fr guiding catheter sometimes cannot be inserted into target arteries (Baba et al., 2014; Sawada et al., 2017). It suggests that the 1.8-Fr tip microballoon catheter have excellent selectability and can be advanced into target vessels less invasively.

Clinical success was also achieved in all cases. The median AML diameter reduction was 34% at a median follow-up of 13 months. Murray et al. reported a mean AML diameter reduction of 39% at a mean follow-up of 39 months. Moreover, it was reported that the tumor shrinkage continues for more than a year (Planche et al., 2011). Thus, the effectiveness of this technique for tumor shrinkage is comparable to the previous reports, although it is affected by the initial volume and tissue composition of the tumor (Hocquelet et al., 2014; Planche et al., 2011).

In our study, ethanol reflux did not occur, and there was no significant decrease in renal function in any patient. In previous reports, renal infarction due to reflux of ethanol occurred in selective TAE using microcatheters in up to 22.5% of cases (Sawada et al., 2017). Thus, it is reasonable to assume that using a microballoon catheter can reduce the risk of renal dysfunction due to the reflux of ethanol. Reducing the risk of renal dysfunction is a significant advantage in the safety of TAE procedure for renal AML because patients with AML often have multiple and bilateral lesions, and sometimes require repeated treatments. Moreover, patients with tuberous sclerosis, which sometimes occurs as a comorbidity with AML, are more likely to present at a younger age, have bilateral lesions, and develop recurrence more often than those without tuberous sclerosis (Kothary et al., 2005; Steiner et al., 1993).

It is known that an ethanol concentration of 70% or more is necessary to denature proteins irreversibly. Therefore, Although there is no consensus on the optimal mixing ratio of ethanol and Lipiodol, 70 to 75% proved sufficient (Hiraki et al., 2009; Kothary et al., 2005; Park et al., 1986; Sawada et al., 2017). The microballoon prevents dilution of ethanol by blood flow, keeping the concentration of ethanol at the desired level in a target vessel. Thus, a microballoon can contribute to increasing the embolization effect of the ethanol mixture.

In this study, pulmonary vasospasm, which is generally a rare complication due to the intravascular administration of ethanol, possibly leading to cardiopulmonary collapse, did not occur. Respiratory complications have been reported to occur in 2% of patients after TAE for AML (Murray et al., 2015). Hiraki et al. reported that pulmonary edema, which is presumed to result from pulmonary vasospasm (Hammer et al., 2001; Mitchell et al., 2006), occurred after TAE for renal AML with lymphangioleiomyomatosis, which sometimes is a comorbid condition with tuberous sclerosis, despite a 4.1 ml (0.07 ml/Kg) ethanol injection (Hiraki et al., 2009). Thus, prevention of pulmonary vasospasm might be required in TAE for renal AML. Most reports of pulmonary vasospasm are cases that occurred after TAE for vascular malformations (Mitchell et al., 2006). Ko et al. reported that the vascular occlusion significantly reduced the elevation of pulmonary artery ethanol concentration and pulmonary artery pressure by reducing the massive washout of ethanol into the systemic artery in TAE for vascular malformations (Ko et al., 2009). Therefore, applying a microballoon catheter in TAE for renal AML can reduce the risk of pulmonary vasospasm by preventing the elevation of pulmonary artery ethanol concentration.

There are some limitations to the present study. First, this study was a retrospective study. Second, the sample size was small. Third, this study included only patients with sporadic renal AML. Renal AMLs in patients with tuberous sclerosis are more likely to recur than those without tuberous sclerosis. Thus, a further study of the effectiveness of this treatment strategy in patients with tuberous sclerosis may be needed.

## Conclusion

Within the limitations of the study, TAE for renal AML using the 1.8-Fr tip microballoon catheter with a mixture of ethanol and Lipiodol is an effective and safe procedure.

## Data Availability

The datasets used and/or analyzed during the current study are available from the corresponding author on reasonable request.

## Abbreviations

AEs: Adverse events
AML: Angiomyolipoma
CT: Computed tomography
CTCAE v5.0: Common Terminology Criteria for Adverse Events version 5.0
DSA: Digital subtraction angiography
eGFR: Estimated glomerular filtration rate
Fr: French
MRI: Magnetic resonance imaging
TAE: Transcatheter arterial embolization
